# Self-rated health over the first five years after stroke

**DOI:** 10.1186/s12883-020-01956-1

**Published:** 2020-10-24

**Authors:** Kerstin Bjälkefur, Salmir Nasic, Eric Bertholds, Katarina Jood, Åsa Rejnö

**Affiliations:** 1 Department of health and social care, Lidköping, Sweden; 2 Research and Development Centre, Skaraborg Hospital, Skövde, Sweden; 3 Tibro Health care centre, Närhälsan Tibro, Sweden; 4 Institute of Neuroscience and Physiology, Department of Clinical Neuroscience, the Sahlgrenska Academy at University of Gothenburg, Gothenburg, Sweden; 5 Department of Neurology, the Sahlgrenska University Hospital, Gothenburg, Sweden; 6 Department of Health Sciences, University West, 461 86 Trollhättan, Sweden; 7 Department of Medicine, Skaraborg Hospital Skövde, Skövde, Sweden

**Keywords:** Longitudinal study, Patient reported outcome measures, Person centered care, Questionnaire, Self-rated health, Sweden

## Abstract

**Background:**

Self-rated health (SRH) focuses on the patient’s own perception, and represents an important patient-reported outcome. The aim was to investigate SRH one to 5 years after stroke, follow the development over time and search for factors associated with SRH.

**Methods:**

Consecutive stroke patients admitted to Stroke Units at the Skaraborg Hospital, Sweden were included 2007–2009 (*n* = 2190). Patient-reported outcomes were collected annually over 5 years using a postal questionnaire. SRH was assessed by the question about general health from SF-36. Factors associated with SRH were investigated by multiple logistic regression analysis.

**Results:**

Response-rate was > 90% at all time points. Overall, 40.2, 41.9, 40.7, 45.0 and 46.3% of the patients reported good SRH, 1 to 5 years after stroke. Performance in activities of daily living (ADL) was strongly associated with good SRH; 49.8 and 14.7% after 1 year in independent and dependent survivors respectively, *p* < 0.001. In independent survivors 1 year after stroke, good SRH was positively associated with female sex (OR = 2.0; *p* = < 0,001), physical activity (OR = 2.14; *p* = < 0,001), car driving (OR = 2.25; *p* = < 0,001), and negatively associated with age (OR = 0.99; *p* = < 0,001), pain (OR = 0.49; *p* = < 0,001), depression (OR = 0.30; *p* = < 0,001), and self-perceived unmet care needs (OR = 0.39; *p* = < 0,001). In dependent survivors, depression (OR = 0.23; *p* = < 0,001) and age (OR = 0.96; *p* = < 0,05), were negatively associated with good SRH 1 year after stroke. Similar patterns were observed throughout the follow-up.

**Conclusion:**

The proportion stroke survivors reporting their health as good is slightly increasing over time. After stroke, SRH is associated with pain, depression, ability to perform activities and self-perceived unmet care needs, indicating that efforts to support stroke survivors in the chronic phase after stroke should concentrate on targeting these factors.

## Background

Onset of stroke is an incident often leading to a major change in life, due to its consequences. Patients often survive with residual impairments and may need support from health services and relatives in the aftermath. Health care and research in stroke has a long tradition of measuring and observing outcome using objective instruments measuring body functions and disability such as the National Institute of Health stroke Scale (NIHSS) and the modified Ranking Scale (mRS). During the last decade research also try to capture the experience of the altered situation of health, i.e. to focus on the patient’s own, subjective perception of health for example through measuring Health-Related Quality of Life (HRQoL) and Self-Rated Health (SRH) [1–5]. Quality of Life (QoL) is an overarching concept by WHO [6] defined as “an individual’s perception of their position in life in the context of the culture and value systems in which they live and in relation to their goals, expectations, standards and concerns”. The concept HRQoL covers the aspects of the overall QoL that are affected by the health status. Self-rated health (SRH) is defined as the persons own rating of the present general health, that is, SRH only focus on the persons rating of their own health. SRH can be assessed both with a single question asking patients to rate their overall health and with a survey questionnaire assessing different dimensions of health such as the Short- Form Health Survey questionnaire (SF-36). This form is a generic multi-dimensional patient-reported survey for patient-reported quality of life. It consists of 36 questions measuring physical and mental health on eight sub scales and it is an example of a widely used instrument for assessing health related quality of life [1, 4]. SRH assessments based on the single question about general health from that form has been shown to be an important indicator of quality of life and an independent predictor of mortality [7–11]. From research with persons newly diagnosed with stroke SRH has been found to be “a multidimensional construct shaped by changes in health status occurring after stroke, individual characteristics and social context” [5]. Given its simplicity based on a single question this might be a favorable way to capture self-perceived health compared to using instruments with several questions.

There are few studies on HRQoL and SRH in stroke survivors beyond the first year after stroke, especially concerning studies with repeated cross-sectional assessments over time. HRQoL and SRH represent important patient-reported long-term outcomes since they focus on the patient’s own perceptions. A review of studies on SRH after stroke [12] states that the diversity in questions and ways to assess SRH makes comparisons of results from different studies difficult. A 6 year follow-up study found that stroke survivors were more dependent and had a lower mean score for general health measured with SF-36 compared to a general population [3]. However, a 10-year follow-up of stroke survivors reported that the majority (62%) rated SRH as excellent, very good or good (women 51%, men 70%) [13] and similar results were also found in a 14-year follow-up study [14]. However, none of these studies have performed repeated follow-ups.

The aim of this study was to explore how a population of unselected stroke patients rated their health one to 5 years after the onset of stroke, to follow the development of SRH over time and to study its correlation to socio-demographic and clinical modifiable factors in order to identify groups at risk for poor SRH after stroke as well as to identify possible modifiable factors.

## Methods

### Study population

Data were obtained from two quality registers assessing stroke care; the Swedish Stroke Register [15], the Skaraborg Longitudinal Stroke Register (SLAG) and the Swedish population registry. The SLAG register has been described in detail elsewhere [16]. In short, SLAG is a local register complementing the Swedish Stroke Register containing data from a postal questionnaire distributed annually over 5 years to all surviving stroke patients admitted to the Stroke Units at the Skaraborg Hospitals from 1 January 2007 to 31 December 2009. For the present investigation we included all patients presenting with a first-ever or recurrent acute ischemic or hemorrhagic stroke as defined according to the World Health Organization criteria [17] at the included stroke units. The population is thus representative for patients cared for at stroke units. Patients with subarachnoid hemorrhage were excluded but not patients with recurrent stroke during the five-year follow-up. Written information about the study and the voluntary nature of participation was given to the participants during hospital stay. No formal written informed consent was obtained, and consent to participate was presumed when a filled out questionnaire was returned. The study was approved by the Regional Ethics Board in Gothenburg (app.nr 270–14).

### Data collection

Information about clinical variables at acute stroke was obtained from the Swedish Stroke Register. These variables included age, sex, first ever stroke, type of stroke, level of consciousness at admission, housing, cohabitation and ability to perform basic activities in daily living (ADL) pre- stroke. Information about patient reported outcomes one to 5 years after stroke were obtained from the SLAG-register. Within the SLAG-project a letter of information about the follow-ups was distributed to the participants along with the postal questionnaire. A reminder was sent if no response to the questionnaire was received after 2 months. A study nurse contacted the respondents by telephone for completing information if the questionnaire was returned incomplete. Information on vital status was obtained by linkage to the Swedish population registry.The postal questionnaire comprised 33 questions with fixed response options. The questions covered for example housing, cohabitation, and degree of dependence in ADL, pain, depression, and social activities. Dependency was defined as needing help with indoor mobility, dressing or toileting, whereas those who were independent in indoor mobility, dressing and toileting were regarded as ADL independent. For social activities the question was “Enter all of the following activities you are able to take part in” with the alternatives” paid work”, “voluntary organizations”, “meet relatives and friends”, and “help relatives and friends”. Patients were considered to be socially active if they had at least one such activity. The annual questionnaire also included questions about physical activity (“How often do you do an exhausting exercise activity” with the response alternatives “never”, “occasionally”, “two to three times a week” and “more than three times a week”), car driving and perceived unmet care needs. The latter included the following items; “Have your needs of home care service been met with respect to: a) health care - help with medication, wound dressing or catheter care, b) service - help with cleaning or grocery shopping, and c) personal care - help with dressing, hygiene, or toileting”, and “Have your needs of rehabilitation or training after stroke been met?”. The response alternatives for the question about unmet care demands were “no needs”, “fulfilled needs”, “partly unmet needs”, “completely unmet needs” and “do not know”. Perceived unmet care needs were defined as perceiving one of these items as partly or completely unmet needs, whereas the others were regarded as perceiving their needs for care as fulfilled. SRH was assessed by the question from the SF-36 questionnaire “In general would you say your health is” with the response alternatives “1=poor, 2=fair, 3=good, 4=very good or 5=excellent”. The five response alternatives for self-rated health were dichotomized by grouping “good” “very good” and” excellent” as good SRH while “poor” and “fair” were categorized as poor SRH.

### Statistics

Descriptive statistics as means and standard deviations for continuous data and frequencies and percentages for categorical data are presented. Mean values and 95% confidence intervals for SRH were calculated and stratified for age and sex for each follow-up. Changes in proportions of patients reporting good SRH over time was tested by the Chi-square test for trend. Univariate analysis (parametric – and non-parametric tests and correlation analysis) were performed to detect variables associated to SRH (data not shown). All variables that were statistically significant at any time-point were in next step included in a multiple logistic regression with “good SRH” as outcome. Thus, multiple logistic regression models were used to explore factors associated with “good SRH” at each follow-up (i.e. cross-sectional analyses) and Odds ratios (OR) with 95% confidence intervals are presented as well as regression parameter (β-coefficient with standard error). Separate models were performed for each follow-up and according to ADL performance. A *p*-value < 0.05 was considered as statistically significant. IBM SPSS v.25 was used to perform statistical analysis.

## Results

**Fig. 1:**
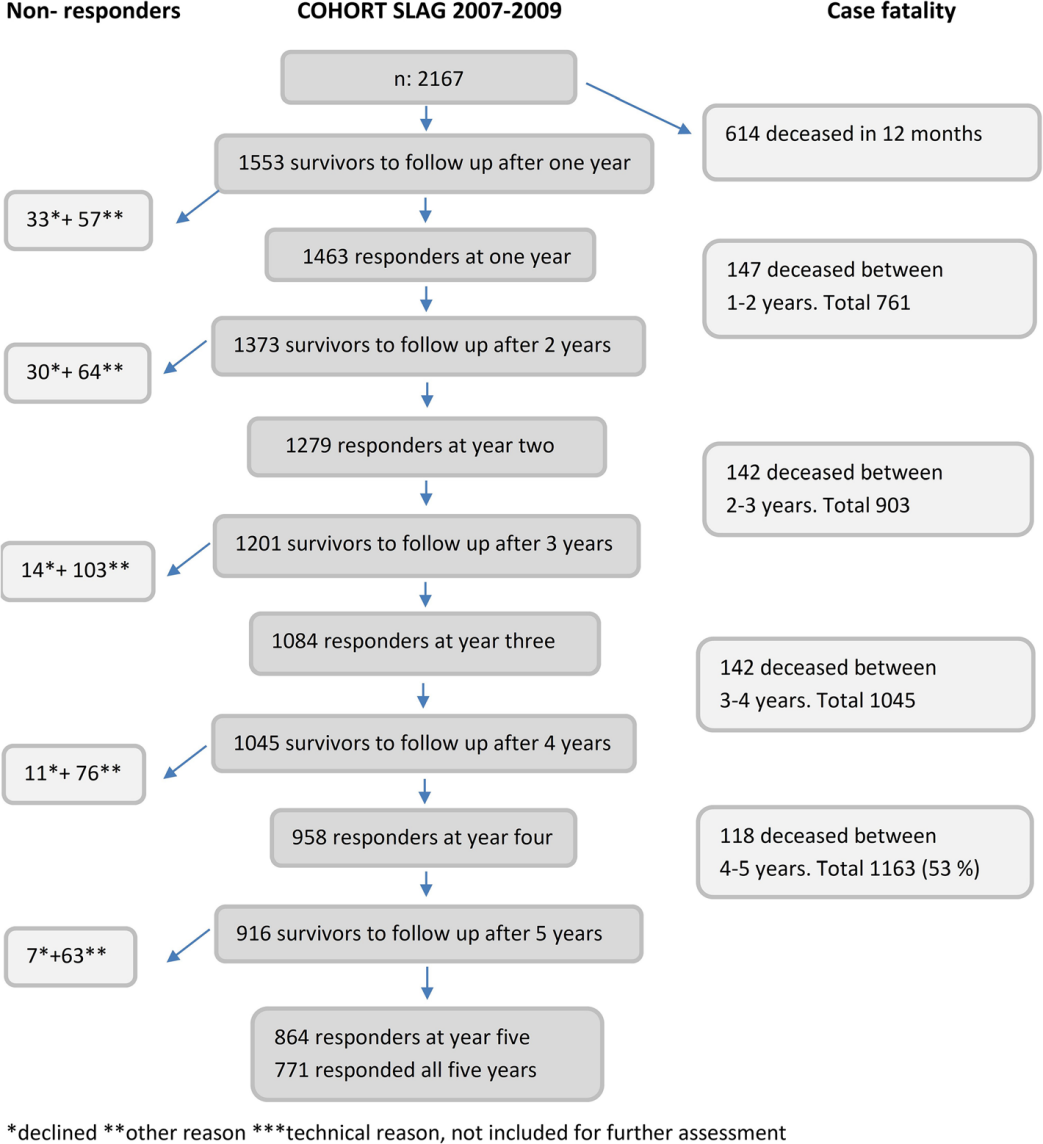
Flow chart showing the number of participants 1–5 years

In total 2167 patients were included of whom 916 (42.3%) were alive at the five-year follow-up (Fig. 1). At each follow-up, all survivors were sent a questionnaire regardless of whether or not they responded to the previous follow-up. The response-rate was > 90% at all time-points.

Descriptive data of the 1553 subjects who were alive at year one after index stroke and invited to take part in the postal survey are given in Table 1. The vast majority (91%) were survivors after an ischemic stroke and one fifth were > 85 years of age at index stroke. One year after stroke 395 (27%) survivors were ADL dependent and 1158 (79%) were living in ordinary housing, of whom 305 (26%) with homecare.

**Table 1:** Descriptive data

	All *n* = 1553
*Age at acute stroke, mean (SD); min-max*	75.3 (10.6); 34–99
*Age group at acute stroke, n* (%)	
< 64 years	247 (16.0)
65–74 years	394 (25.5)
75–84 years	586 (37.9)
≥ 85 years	320 (20.7)
*Sex, n (%)*	
Male	878 (56.8)
Female	669 (43.2)
*First ever stroke, n (%)*	1179 (76.3)
*Type of stroke, n (%)*	
Ischemic stroke (ICD code I63)	1414 (91.4)
Hemorrhagic stroke (ICD code I61)	126 (8.1)
Unspecified (ICD code I64)	7 (0.5)
*Level of consciousness at acute stroke n (%)*	
Fully awake	1423.(92.2)
Drowsy/unconscious	120 (7.8)
*Living alone pre stroke (%)*	721 (46.6)
*Living alone at 1 year after stroke (%)*	746 (47.9)
*Housing situation pre stroke n (%)*	
Ordinary housing	1238 (80)
Ordinary housing with home care	225 (14.5)
Skilled nursing home	80 (5.2)
Missing data	4 (0.3)
*Housing situation 1 year after stroke n (%)*	
Ordinary housing	853 (54.8)
Ordinary housing with home care	305 (19.6)
Skilled nursing home	301 (19.3)
Missing data	94 (6)
*ADL-independent before stroke (%)*	1422 (91.9)
*ADL-independent 1 year after stroke (%)*	1051 (67.5)

*ICD* International classification of diseases; *ADL* Activity of daily living

**Fig. 2:**
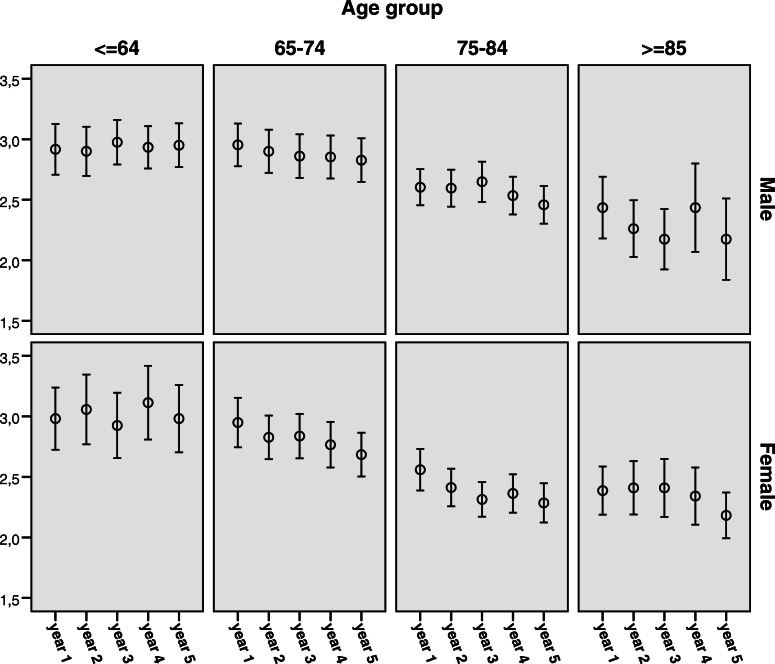
Change in self-rated health over time among those who survived the entire follow-up. Mean*with 95% confidence interval at each follow- up.*Self-Reported Health; 1 = poor, 2 = fair, 3 = good, 4 = very good, and 5 = excellent health

Overall, in repeated *cross-sectional analysis*, the proportion of survivors reporting good SRH increased slightly during follow-up with 40.2, 41.9, 40.7, 45.0 and 46.3% reporting good SRH one to 5 years after stroke, *p*-value (for trend) =0.002. At all time-points SRH was negatively correlated to age (p-value < 0.001 all through)*.* However, in *longitudinal analysis* of those who survived the entire follow-up, younger survivors rated their SRH relatively stable during follow-up, whereas older survivors worsened. This pattern was observed for both men and women (Fig. 2).

In cross-sectional analysis, independence in ADL was strongly associated with good SRH; the proportion-rating good SRH was 49.8 and 56.8% in ADL-independent, and 14.7 and 16.6% in ADL-dependent survivors, one and 5 years after stroke, respectively (*p* < 0.001 for both). Therefore, when exploring factors associated with SRH we divided the population with respect to ADL-dependency. In a multiple model of ADL-independent stroke survivors, female sex and physical activity showed consistent positive associations with good SRH, whereas age, pain, depression and perceived unmet care were constantly negatively associated with good SRH (Table 2 and supplemental table1). Car driving was independently associated with good SRH during the first 2 years after stroke, but not during the late part of follow-up.

**Table 2:** Cross-sectional analysis; factors associated with “good self-rated health” 1–5 years after stroke in ADL-independent survivors. Odds ratios

	Year 1 (*n* = 1051)	Year 2 (*n* = 911)	Year 3 (*n* = 780)	Year 4 (*n* = 718)	Year 5 (*n* = 632)
	OR with 95% CI	OR with 95% CI	OR with 95% CI	OR with 95% CI	OR with 95% CI
*Age (years)*	0.98** (0.96–0.99)	0.97** (0.95–0.99)	0.97** (0.95–0.99)	0.96** (0.94–0.98)	0.99 (0.97–1.01)
*Female*	2.0** (1.41–2.84)	1.98** (1.33–2.94)	1.64* (1.06–2.53)	2.34** (1.48–3.72)	1.23 (0.78–1.95)
*Living alone*	1.27 (0.90–1.79)	1.00 (0.68–1.46)	1.68* (1.07–2.62)	0.67 (0.43–1.03)	0.82 (0.52–1.29)
*Housing situation*					
Ordinary housing	ref.	ref.	ref.	ref.	ref.
Ordinary housing with homecare	0.88 (0.56–1.38)	1.06 (0.62–1.82)	0.40** (0.22–0.74)	1.45 (0.77–2.71)	0.50* (0.26–0.998)
Nursing home	1.72 (0.82–3.64)	1.29 (0.55–3.06)	0.49 (0.15–1.64)	2.29 (0.64–8.17)	1.47 (0.46–4.71))
*Pain*					
Never	ref.	ref.	ref.	ref.	ref.
Sometimes	0.49** (0.36–0.68)	0.51** (0.36–0.74)	0.42** (0.28–0.65)	0.40** (0.26–0.61)	0.40** (0.25–0.63)
Often or constantly	0.18** (0.11–0.28)	0.15** (0.09–0.24)	0.07** (0.04–0.13)	0.10** (0.05–0.18)	0.08** (0.04–0.15)
*Depression*					
Never	ref.	ref.	ref.	ref.	ref.
Sometimes	0.30** (0.21–0.41)	0.29** (0.20–0.42)	0.22** (0.14–0.34)	0.36** (0.23–0.56)	0.39** (0.25–0.60)
Often or constantly	0.14** (0.08–0.26)	0.16** (0.08–0.31)	0.13** (0.06–0.29)	0.08** (0.03–0.18)	0.10** (0.03–0.25)
*Physical activity*					
Never or not applicable	ref.	ref.	ref.	ref.	ref.
Sporadic	1.59* (1.06–2.39)	1.61* (1.02–2.54)	1.56 (0.95–2.58)	2.08** (1.25–3.46)	1.36 (0.80–2.30)
2–3 times a week or more	2.14** (1.47–3.13)	1.84** (1.19–2.86)	3.79** (2.33–6.17)	4.05** (2.39–6.85)	2.48** (1.46–4.21)
*At least one social activity a week*	1.42 (0.75–2.69)	1.64 (0.79–3.40)	1.73 (0.78–3.80)	1.38 (0.56–3.38)	2.32 (0.88–6.06)
*Perceived unmet care needs*	0.39** (0.27–0.57)	0.24** (0.15–0.37)	0.49** (0.30–0.78)	0.36** (0.22–0.59)	0.32** (0.19–0.54)
*Driving car*	2.25** (1.40–3.62)	2.45** (1.40–4.27)	1.67 (0.88–3.15)	1.68 (0.85–3.33)	0.99 (0.48–2.07)

Cross-sectional multivariable logistic regression analysis. ADL indicates activities in daily living* < 0.05, ** < 0.01, ref. = reference category. Good self-rated health = the response alternatives “good”, “very good” and” excellent” grouped together

In ADL- dependent survivors, age, depression and pain showed a consistent negative association with good SRH during follow-up. At least one social activity and perceived unmet care needs also showed association with good SRH, with a positive association for social activity and negative for unmet care needs (Table 3 and supplemental table 2). However, although in the same direction throughout the follow-up, these associations reached the level of statistical significance only at two of the five time points.

**Table 3:** Cross-sectional analysis; factors associated with “good self-rated health” 1–5 years after stroke in ADL-dependent survivors. Odds ratios

	Year 1 (*n* = 395)	Year 2 (*n* = 355)	Year (*n* = 310)	Year 4 (*n* = 255)	Year 5 (*n* = 231)
	OR with 95% CI	OR with 95% CI	OR with 95% CI	OR with 95% CI	OR with 95% CI
*Age (years)*	0.96* (0.92–0.99)	1.00 (0.97–1.04)	0.99 (0.94–1.04)	0.95 (0.90–1.00)	0.95 (0.90–1.01)
*Female*	0.67 (0.32–1.42)	0.88 (0.43–1.80)	1.01 (0.40–2.54)	0.72 (0.26–1.98)	0.78 (0.26–2.27)
*Living alone*	1.01 (0.35–2.90)	2.40 (0.86–6.64)	1.51 (0.49–4.62)	0.94 (0.29–3.06)	1.46 (0.45–4.75)
*Housing situation*					
Ordinary housing	ref.	ref.	ref.	ref.	ref.
Ordinary housing with homecare	1.08 (0.34–3.44)	2.54 (0.68–9.42)	0.88 (0.24–3.30)	1.13 (0.24–5.30)	1.77 (0.33–9.56)
Nursing home	1.32 (0.31–5.63)	0.91 (0.19–4.34)	0.38 (0.08–1.83)	2.58 (0.43–15.51)	0.64 (0.09–4.35)
*Pain*					
Never	ref	ref.	ref.	ref.	ref.
Sometimes	0.78 (0.36–1.67)	0.60 (0.29–1.24)	0.65 (0.28–1.53)	0.72 (0.26–1.97)	0.15** (0.05–0.44)
Often or constantly	0.63 (0.26–1.54)	0.35* (0.13–0.94)	0.12* (0.03–0.61)	0.25* (0.07–0.98)	0.06** (0.01–0.29)
*Depression*					
Never	ref	ref.	ref.	ref.	ref.
Sometimes	0.23** (0.11–0.50)	0.44* (0.20–0.98)	1.08 (0.43–2.72)	0.30* (0.10–0.92)	0.30* (0.10–0.88)
Often or constantly	0.10** (0.03–0.29)	0.13** (0.04–0.40)	0.13 (0.01–1.17)	0.18* (0.03–0.96)	0.20* (0.04–0.91)
*Physical activity*					
Never or not applicable	ref.	ref.	ref.	ref.	ref.
Sporadic	1.30 (0.53–3.19)	0.74 (0.23–2.39)	3.77 * (1.21–11.75)	1.87 (0.48–7.24)	0.58 (0.13–2.65)
2–3 times a week or more	1.52 (0.49–4.72)	2.07 (0.78–5.48)	2.46 (0.66–9.17)	3.06 (0.81–11.48)	0.92 (0.20–4.18)
*At least one social activity a week*	2.46 (0.81–7.50)	1.64 (0.68–3.93)	8.84* (1.11–70.22)	7.65** (1.78–32.79)	1.14 (0.35–3.70)
*Perceived unmet care needs*	0.79 (0.41–1.55)	0.66 (0.35–1.27)	0.27** (0.11–0.67)	0.24* (0.09–0.62)	1.10 (0.40–2.86)
*Driving car*	3.87 (0.63–23.96)	1.61 (0.12–21.86)	n.a.	n.a.	n.a.

Cross-sectional multivariable logistic regression analysis. ADL indicates activities in daily living* < 0.05, ** < 0,01 ref. = reference category. n.a. =not applicable, *n* < 5 in any subgroup.” Good self-rated health = the response alternatives “good”, “very good” and” excellent” grouped together

## Discussion

This study analyses SRH and its development over time after stroke, in an unselected hospital-based cohort of stroke patients who were followed annually for 5 years. All through follow-up, about four of ten stroke survivors reported good SRH, with a slight increase over time. This increase might partly be explained by death of the most dependent patients, leaving proportionally more patients with less disabilities and dependencies alive during the entire follow-up. These patients are probably more prone to report their health as good since the most important factor for a good SRH was ADL performance.

We found pain and depression to be consistently independently associated with SRH over time, in both ADL dependent and independent survivors. In addition, among independent stroke survivors, SRH was independently associated with female sex, physical activity, car driving, and perceived unmet care needs, while social activity and to a lesser extent perceived unmet care needs were the additional associated factors among the ADL dependent survivors.

A municipality-based study of 187 survivors of first ever stroke, SRH was investigated using similar but not identical response alternatives compared to the present study, and found that 62% of the participants rated their health as good or rather good at 3 months and 78% at 12 months [7]. In a population-based study with 145 stroke survivors 10 years after stroke [13] good SRH was reported by 62%, with a higher proportion in men (70%) than in women (51%). Compared to our study, both studies [7, 13] have only included patients with a first-ever stroke, which may contribute to the better outcome in their samples. A 14 year follow-up study including all patients still alive at the time of the follow-up (regardless of recurring stroke or not) found that about half of the men ≤64 years as well as women of all ages rated their health as good, the exception was men ≥65 years where the proportion only were 25%. When taking the response alternatives “good” and “satisfactory” together no such gender difference were found.

Depression and pain were strong negative predictors of SRH all through follow-up, both among independent and dependent survivors. The negative impact of both pain and depression in the aftermath of stroke is well known [18]. It has also been shown that despite affecting quality of life, limiting activities in daily life and participation in social activities are this often not recognized by health care professionals [18]. If health- and social care pay more attention to optimize the assessment of these symptoms and treat them, it could lead to improved SRH and increased degree of well-being. Thus, increased attention to pain and depression among survivors at long-term after stroke is warranted regardless of the degree of disability. Interventions may include medication as well as increased physical- and social activity.

In the ADL-independent group, we found that women rated their health better than men. This is an unusual finding for both a general population [19] and a stroke population [8, 20] but have been reported previously for women ≥65 [14]. The finding in present study was disclosed when analyzing dependent and independent survivors separately. Our finding indicates that there may be gender differences in the perception of health among survivors with mild disability after stroke. Possibly, higher expectations among men to resume more demanding activities could be one explanation, as it has been shown that men’s expectations of their own ability and activity in society is an important part of traditional masculinity [21].

Physical activity and driving a car were both strong predictors of SRH among independent stroke survivors, while no or weaker associations were observed among ADL dependent survivors, probably reflecting that the higher degree of disability in the latter group hampers these more demanding activities. Instead, in this group our results indicate an association between SRH and the possibility to take part in social activities, which was not observed among the independent survivors. Car driving along with walking ability and a social network have been found as a long-term predictors of social activity in age groups < 75 years [22]. The results of the present study indicate that the possibility to perform and take part in activities is an important key for SRH after stroke and that strategies to support and increase social and physical activity modified to the individual’s degree of disability will be beneficial for the perceived health. This stresses the importance of supporting mobility after stroke.

We also found that perceived unmet care needs was negatively associated with SRH. This is an interesting observation, as this factor may be modifiable. The association was stronger and more consistent over time among ADL independent stroke survivors, indicating needs in this group that are neglected by health care and social support. Previous studies on unmeet care needs have typically not studied the correlation to SRH but rather found that unmeet care needs often belong to the health dimension [23, 24]. The type of unmet care needs that was most frequently reported in the present study was the need of rehabilitation (23%).

It has been shown that perception of SRH besides physical limitations is influenced both by previous experiences of ill health and views on recovery in the future [5]. This emphasizes the need of conveying realistic optimism and hope about the patients’ recovery. However, further studies, including qualitative interview studies, are needed to further explore the individuals’ perception of care needs after stroke.

### Methodological considerations

The strengths of this study include the relatively large cohort of consecutive patients with acute stroke, discharged from all stroke-units serving the geographical area. According to national guidelines in Sweden, all patients with acute stroke should be referred to stroke unit care at a hospital without delay [25]. Thus, despite being a hospital-based sample, it is fairly representative for the entire stroke population. Moreover, the response rate was high, the proportion of participants lost at follow-up was low and we used a simple validated instrument for SRH. The longitudinal design with repeated measures over 5 years means that we can identify factors consistently associated with SRH in a long-term perspective and study development and changes over time.

Although most factors found to be associated with reporting health as good were fairly stable over time, all associations were not consistent. Given the limited sample size the differences found between the years could be a matter of limited statistical power for some variables at some years but the differences could also imply that the importance of some variables change over time. However, when statistical significance with consistent results is achieved at more than one cross-sectional model (1 year) it may suggest a more consolidated association between the explored variable and SRH as outcome. Limitations include not limiting the participants to only first-ever stroke and that we do not have data on recurrent stroke during the five-year follow-up. Also the relatively small number of patients dependent in ADL and that the assessment of factors related to SRH was restricted to a number of variables chosen for the postal questionnaire can be seen as limitations. Thus, we cannot exclude that some other factors, not asked for, may be important determinants of SRH after stroke. We did not use validated instruments for the assessment of depression, pain, and activities. However, this choice was made to enable coverage of many areas and still keep the questionnaire short and easy for the respondents to fill out, which probably contributed to the high response rate.

## Conclusion

The proportion stroke survivors reporting their health as good is slightly increasing over time. The proportion of independent survivors rating their health as good is more than three times the proportion of dependent survivors. Performance in activities of daily living (ADL) is hence strongly associated with good SRH. Self-rated health is also associated with pain, depression, ability to perform physical and social activities and self-perceived unmet care needs. Our findings indicate that efforts to support stroke survivors in the chronic phase after stroke should concentrate on targeting the aforementioned factors. Moreover, the impact of unmet care needs is most prominent in those independent after stroke, indicating that needs in this group may be a “blind spot” for health care professionals and not attended to in the same way as for persons dependent in ADL, where needs are more evident. Further, factors related to gender, such as different ways of valuing personal independence, may also be of importance, thus emphasizing a person centered approach in care.

## Supplementary information

**Additional file 1: Table S1** Cross-sectional analysis; factors associated with “good self-rated health” 1–5 years after stroke in ADL-independent survivors. Regression parameters. **Table S2** Cross-sectional analysis; factors associated with “good self-rated health” 1–5 years after stroke in ADL-dependent survivors. Regression parameters

## Data Availability

The datasets used and/or analysed during the current study are available from the corresponding author on reasonable request. The data are not publicly available due to privacy or ethical restrictions.

## Abbreviations

ADL: Activities of Daily Living
CI: confidence interval
HRQoL: Health-Related Quality of Life
ICD: International classification of diseases
mRS: modified Ranking Scale
n.a: not applicable
NIHSS: National Institute of Health stroke Scale
OR: Odds Ratio
ref: reference category
SD: standard deviation
SF-36: The Short- Form Health Survey questionnaire
SLAG: Skaraborg Longitudinal Stroke Register
SPSS: Statistical Package for the Social Sciences
SRH: Self-Rated Health
