# Supplementary material for: Self-rated health over the first five years after stroke
Source: BMC Neurol. 2020 Oct 24;20:389. doi: 10.1186/s12883-020-01956-1 (PMC7585295; doi:10.1186/s12883-020-01956-1)
Supplement: Supplementary file 1 — Additional file 1: Table S1 Cross-sectional analysis; factors associated with “good self-rated health” 1–5 years after stroke in ADL-independent survivors. Regression parameters. Table S2 Cross-sectional analysis; factors associated with “good self-rated health” 1–5 years after stroke in ADL-dependent survivors. Regression parameters [file 12883_2020_1956_MOESM1_ESM.docx]

**Supplemental table 1**. Cross-sectional analysis; factors associated with “good self-rated health” 1-5 years after stroke in ADL-independent survivors. Regression parameters.

|  | Year 1 (n=1051) | Year 2 (n=911) | Year 3 (n=780) | Year 4 (n=718) | Year 5 (n=632) |
| --- | --- | --- | --- | --- | --- |
|  | **β-coefficient (S.E)** | **β-coefficient (S.E)** | **β-coefficient (S.E)** | **β-coefficient (S.E)** | **β-coefficient (S.E)** |
| *Age (years)* | -0.023 (0.008)** | -0.028 (0.010)** | -0.031 (0.010)** | -0.039 (0.012)** | -0.009 (0.012) |
| *Female* | 0.692 (0.179)** | 0.684 (0.201)** | 0.495 (0.222)* | 0.851 (0.236)** | 0.207 (0.235) |
| *Living alone* | 0.240 (0.175) | 0.001 (0.194) | 0.517 (0.229)* | -0.407 (0.225) | -0.194 (0.229) |
| *Housing situation* |  |  |  |  |  |
| Ordinary housing | ref. | ref. | ref. | ref. | ref. |
| Ordinary housing  with homecare | -0.129 (0.231) | 0.058 (0.275) | -0.910 (0.313)** | 0.371 (0.320) | -0.682 (0.347)* |
| Nursing home | 0.545 (0.381) | 0.258 (0.439) | -0.707 (0.613) | 0.827 (0.650) | 0.389 (0.592) |
| *Pain* |  |  |  |  |  |
| Never | ref. | ref. | ref. | ref. | ref. |
| Sometimes | -0.704 (0.166)** | -0.666 (0.187)** | -0.862 (0.217)** | -0.918 (0.220)** | -0.913 (0.234)** |
| Often or constantly | -1.742 (0.239)** | -1.930 (0.264)** | -2.640 (0.324)** | -2.344 (0.318)** | -2.515 (0.320)** |
| *Depression* |  |  |  |  |  |
| Never | ref. | ref. | ref. | ref. | ref. |
| Sometimes | -1.218 (0.167)** | -1.237 (0.188)** | -1.518 (0.222)** | -1.025 (0.223)** | -0.951 (0.227)** |
| Often or constantly | -1.939 (0.301)** | -1.856 (0.343)** | -2.003 (0.394)** | -2.580 (0.441)** | -2.447 (0.544)** |
| *Physical activity* |  |  |  |  |  |
| Never or not applicable | ref. | ref. | ref. | ref. | ref. |
| Sporadic | 0.466 (0.206)* | 0.479 (0.232)* | 0.447 (0.256) | 0.733 (0.260)** | 0.306 (0.270) |
| 2-3 times a week or more | 0.763 (0.193)** | 0.612 (0.223)** | 1.333 (0.249)** | 1.398 (0.269)** | 0.909 (0.270)** |
| *At least one social activity a week* | 0.351 (0.325) | 0.497 (0.371) | 0.547 (0.403) | 0.321 (0.458) | 0.841 (0.491) |
| *Perceived unmet care needs* | -0.929 (0.189)** | -1.443 (0.233)** | -0.717 (0.240)** | -1.011 (0.250)** | -1.134 (0.262)** |
| *Driving car* | 0.813 (0.242)** | 0.895 (0.284)** | 0.512 (0.324) | 0.520 (0.349) | -0.006 (0.375) |

Cross-sectional multivariable logistic regression analysis. ADL indicates activities in daily living*<0.05, ** <0.01, ref=reference category. Good self-rated health=the response alternatives “good”, “very good” and ”excellent” grouped together.

**Supplemental table 2**. Cross-sectional analysis; factors associated with “good self-rated health” 1-5 years after stroke in ADL-dependent survivors. Regression parameters.

|  | Year 1 (n=395) | Year 2 (n=355) | Year (n=310) | Year 4 (n=255) | Year 5 (n=231) |
| --- | --- | --- | --- | --- | --- |
|  | **β-coefficient (S.E)** | **β-coefficient (S.E)** | **β-coefficient (S.E)** | **β-coefficient (S.E)** | **β-coefficient (S.E)** |
| *Age (years)* | -0.041(0.021)* | 0.005 (0.019) | -0.014 (0.025) | -0.048 (0.027) | -0.051 (0.030) |
| *Female* | -0.396 (0.381) | -0.124 (0.362) | 0.010 (0.471) | -0.329 (0.517) | -0.253 (0.548) |
| *Living alone* | 0.011 (0.538) | 0.874 (0.520) | 0.411 (0.571) | -0.059 (0.602) | 0.378 (0.603) |
| *Housing situation* |  |  |  |  |  |
| Ordinary housing | ref. | ref. | ref. | ref. | ref. |
| Ordinary housing with homecare | 0.076 (0.592) | 0.931 (0.669) | -0.123 (0.673) | 0.126 (0.786) | 0.575 (0.859) |
| Nursing home | 0.283 (0.737) | -0.092 (0.796) | -0.969 (0.803) | 0.948 (0.915) | -0.451 (0.980) |
| *Pain* |  |  |  |  |  |
| Never | ref | ref. | ref. | ref. | ref. |
| Sometimes | -0.249 (0.389) | -0.504 (0.366) | -0.425 (0.433) | -0.329 (0.515) | -1.902 (0.550)** |
| Often or constantly | -0.467 (0.458) | -1.050 (0.505)* | -2.093 (0.813)* | -1.365 (0.689)* | -2.866 (0.829)** |
| *Depression* |  |  |  |  |  |
| Never | ref | ref. | ref. | ref. | ref. |
| Sometimes | -1.464 (0.393)** | -0.813 (0.403)* | 0.080 (0.470) | -1.206 (0.574)* | -1.200 (0.544)* |
| Often or constantly | -2.435 (0.614)** | -2.072 (0.595)** | -2.068 (1.136) | -1.732 (0.865)* | -1.591 (0.765)* |
| *Physical activity* |  |  |  |  |  |
| Never or not applicable | ref. | ref. | ref. | ref. | ref. |
| Sporadic | 0.264 (0.457) | -0.302 (0.599) | 1.327 (0.580) * | 0.628 (0.690) | -0.534 (0.770) |
| 2-3 times a week or more | 0.418 (0.578) | 0.728 (0.497) | 0.899 (0.672) | 1.118 (0.675) | -0.079 (0.770) |
| *At least one social activity a week* | 0.902 (0.568) | 0.492 (0.447) | 2.179 (1.058)* | 2.034 (0.743)** | 0.133 (0.600) |
| *Perceived unmet care needs* | -0.230 (0.340) | -0.408 (0.330) | -1.292 (0.456)** | -1.446 (0.491)* | 0.062 (0.505) |
| *Driving car* | 1.355 (0.930) | 0.478 (1.330) | n.a. | n.a. | n.a. |

Cross-sectional multivariable logistic regression analysis. ADL indicates activities in daily living*<0.05, ** <0,01 ref=reference category. n.a. =not applicable, n< 5 in any subgroup. ”Good self-rated health=the response alternatives “good”, “very good” and ”excellent” grouped together.
